# A New Approach to Produce HIV-1 Envelope Trimers: BOTH CLEAVAGE AND PROPER GLYCOSYLATION ARE ESSENTIAL TO GENERATE AUTHENTIC TRIMERS*

**DOI:** 10.1074/jbc.M115.656611

**Published:** 2015-06-18

**Authors:** Wadad AlSalmi, Marthandan Mahalingam, Neeti Ananthaswamy, Christopher Hamlin, Dalia Flores, Guofen Gao, Venigalla B. Rao

**Affiliations:** From the Department of Biology, The Catholic University of America, Washington, D. C. 20064

**Keywords:** AIDS, glycosylation, human immunodeficiency virus (HIV), recombinant protein expression, vaccine, envelope protein, gp140 trimer

## Abstract

The trimeric envelope spike of HIV-1 mediates virus entry into human cells. The exposed part of the trimer, gp140, consists of two noncovalently associated subunits, gp120 and gp41 ectodomain. A recombinant vaccine that mimics the native trimer might elicit entry-blocking antibodies and prevent virus infection. However, preparation of authentic HIV-1 trimers has been challenging. Recently, an affinity column containing the broadly neutralizing antibody 2G12 has been used to capture recombinant gp140 and prepare trimers from clade A BG505 that naturally produces stable trimers. However, this antibody-based approach may not be as effective for the diverse HIV-1 strains with different epitope signatures. Here, we report a new and simple approach to produce HIV-1 envelope trimers. The C terminus of gp140 was attached to Strep-tag II with a long linker separating the tag from the massive trimer base and glycan shield. This allowed capture of nearly homogeneous gp140 directly from the culture medium. Cleaved, uncleaved, and fully or partially glycosylated trimers from different clade viruses were produced. Extensive biochemical characterizations showed that cleavage of gp140 was not essential for trimerization, but it triggered a conformational change that channels trimers into correct glycosylation pathways, generating compact three-blade propeller-shaped trimers. Uncleaved trimers entered aberrant pathways, resulting in hyperglycosylation, nonspecific cross-linking, and conformational heterogeneity. Even the cleaved trimers showed microheterogeneity in gp41 glycosylation. These studies established a broadly applicable HIV-1 trimer production system as well as generating new insights into their assembly and maturation that collectively bear on the HIV-1 vaccine design.

## Teaser

**Background:** HIV-1 envelope trimer is a candidate for designing an effective HIV vaccine.

**Results:** gp140 attached to Strep-tag through a long linker is used to purify HIV trimers. Cleaved, uncleaved, and fully and partially glycosylated trimers are characterized.

**Conclusion:** Cleaved and glycosylated gp140 assembles into authentic propeller-shaped trimers.

**Significance:** This system could generate HIV-1 trimers for clinical trials and vaccine manufacture.

## Introduction

AIDS, caused by HIV-1, is a global epidemic. More than 30 million people worldwide currently live with HIV infection, and nearly 2 million people die of AIDS every year. Nine genetic subtypes and numerous circulating recombinant forms have been identified. Coupled with this diversity is the extraordinary evolution of the viral envelope protein (Env) in response to host immune pressures. Designing an Env immunogen that can stimulate antibodies (Abs),^2^ which in turn can block entry of genetically diverse HIV-1 viruses, has remained as the “holy grail” of the HIV vaccine field (1, 2).

The trimeric Env spike of the HIV-1 virion is the virus entry machine. It is a trimer of heterodimers composed of the glycoproteins gp120 and gp41 produced by cleavage of the precursor protein gp160 (3, 4). Entry involves a series of well orchestrated interactions between these proteins and the receptor molecules present on the target cell (5). The first step might be the capture of the virus through interactions between the V1V2 domain of gp120 and a surface molecule, such as the α4β7 integrin of the mucosal T lymphocytes (6, 7). This might bring the virus into close proximity to CD4, the primary receptor. Binding to CD4 causes a conformational change in gp120, exposing a site in the V3 domain that binds to the chemokine co-receptor CCR5 or CXCR4 (8–13). A series of conformational changes ensue, resulting in the insertion of the gp41 fusion peptide into the host cell membrane (14). The viral lipid bilayer fuses with the plasma membrane, releasing the nucleocapsid core into the target cell (15). Therefore, Env-specific Abs that can interfere with any of the steps common to diverse HIV-1 viruses can prevent transmission of HIV into the host.

Several human monoclonal Abs (mAbs), referred to as broadly neutralizing Abs (BnAbs), have been discovered that can neutralize infection of a large spectrum of genetically diverse HIV-1 viruses. These include, for instance, BnAbs b12 and VRC01, which bind to the CD4 binding site of gp120; 2F5 and 4E10, which bind to the membrane-proximal external region (MPER) of gp41; and PG9 and PG16, which bind to the V1V2 domains of the trimer (16–19). Most of these Abs recognize conformational epitopes and are produced either by “elite controller” individuals with chronic HIV infections or by selection of rare B cell clones present in HIV-1-infected individuals (20). They also exhibit unusual features, such as the presence of a long heavy chain 3 complementarity-determining region covering a large area of the epitope as well as dozens of somatic mutations introduced by a process known as “affinity maturation” driven by the evolving envelope protein (21). Attempts to induce such BnAbs in animal models or in humans by vaccination with recombinant Env immunogens have thus far failed (22–25).

One reason for this failure may be that the subunit Env immunogens do not recapitulate the trimeric structure of the native Env spike present on the HIV-1 virion (26). It has been hypothesized that exposure to “native” trimers could lead to activation and expansion of rare B cell clones of the right BnAb lineage (27). Furthermore, such a trimer can also be used as a scaffold to engineer variants that represent a common structure present in diverse HIV-1 strains. However, production of Env trimers that mimic the native spike has remained a challenge, in part because the recombinant trimers either are unstable or aggregate. Recently, Ringe *et al.* (26) discovered that an HIV-1 subtype A isolate BG505 naturally produces relatively stable trimers. By further stabilizing the trimer with mutations that cross-link cleaved gp120 and gp41 through a disulfide bond (SOSIP), they could produce “native-like” trimers. These were then captured by the BnAb 2G12 and purified (26, 28). The structures of the trimers complexed with various BnAbs have been determined by cryo-EM and x-ray crystallography (29, 30). However, the Ab-based approach is not as effective with diverse HIV-1 strains that might differ in the epitope signature. For instance, the wild-type BG505 gp140 was mutated by changing Thr-332 to Asn to create the epitope binding site for 2G12 (26, 31). It is, however, possible, in principle, to use a trimer-specific BnAb, such as PGT145, to selectively capture the trimers from diverse HIV-1 strains (32).

Our laboratory has been investigating the design of HIV-1 Env immunogens and efficient vaccine delivery systems (33–35). Here, we report a new system to isolate and characterize Env trimers, potentially from any HIV-1 virus strain. First, we show that by attaching a highly specific 8-amino acid (aa) Strep-tag II separated from the C terminus of gp140 by a long ∼20-aa linker, the Env protein can be efficiently captured by Strep-Tactin directly from the culture supernatant. The bound protein can then be dissociated under mild conditions to generate ∼95% pure Env in a single step. Second, a screening strategy was developed to optimize any Env recombinant construction for maximal trimer production. The JRFL Env gp140 selected by this approach produced ∼70% of gp140 as trimers. Third, the cleaved JRFL Env trimers exhibited the classic three-blade propeller shape (36), and their biochemical and antigenic properties are consistent with the native trimers. Fourth, we found that both cleavage and proper glycosylation are critical for maturation of gp140 into authentic trimers. Although gp140 could trimerize without cleavage, uncleaved trimers entered aberrant pathways, generating hyperglycosylated and conformationally heterogeneous particles. Finally, the trimers, including the cleaved propeller trimers, showed microheterogeneity in the extent of gp41 glycosylation. These studies established a broadly applicable system for production and characterization of HIV-1 trimers and generated new insights into the assembly and maturation of HIV-1 trimers that will have implications for the design of an effective HIV vaccine.

## Materials and Methods

### 

#### 

##### Antibodies

The following reagents were obtained through the National Institutes of Health AIDS Reagent Program, Division of AIDS, NIAID: HIV-1 gp120 monoclonal antibody (2G12) (37–41) from Dr. Hermann Katinger, HIV-1 gp120 mAb (VRC01) (17) from Dr. John Mascola, PGT 121 (catalog no. 12343) (42), HIV-1 gp41 monoclonal antibody (F240) (43), and HIV-1 gp120 monoclonal antibody (F105) (44–47) from Dr. Marshall Posner and Dr. Lisa Cavacini. The PG9 (19), PG16 (19), PGT145 (42), PGT151 (48), and b6 (16) were obtained from the Scripps Research Institute and International AIDS Vaccine Initiative Neutralizing Antibody Center. Polyclonal Abs against HIV-1 JRFL gp140 were raised in mice in our laboratory.

##### Clone Constructions

The furin-expressing plasmid, Furin:FLAG/pGEM7Zf(+), was obtained from Dr. Gary Thomas (Vollum Institute, Portland, OR). The furin fragment from this plasmid was subcloned into pcDNA3.1(−) (Life Technologies, Inc.) using EcoRI and HindIII restriction sites.

Codon-optimized gp140 DNAs from JRFL-FD, SF162-FD, and CONPEP-FD were provided by Dr. Peter Kwong (Vaccine Research Center, National Institutes of Health). These DNAs contained the sequence corresponding to gp120 and gp41 ectodomain up to aa 683. In addition, they have the human CD5 secretion signal at the 5′-end, furin cleavage-resistant mutation SEKS at the junction of gp120 and gp41, and FD followed by the hexahistidine tag at the C terminus (49). Using the JRFL-FD as the starting template, a series of additional mutations were introduced. These include, for instance, SOSIP mutations (50, 51), stabilizing mutations (52), enhanced furin cleavage site RRRRRR (53), and various truncations shown in Fig. 2 and described under “Results.” The JRFL gp120 clone was also constructed from the same template by PCR amplification of the appropriate sequence corresponding to gp120.

The BG505 (BG505.W6M.ENV.C2) (28, 54) gp140 envelope sequence was codon-optimized and the optimized sequence was synthesized using the GenArt Strings technology (Life Technologies). During this process, a series of mutations were also introduced, as follows: Asn at aa 332 to introduce an *N*-linked glycosylation site that allows binding of BG505 gp140 to 2G12 (55) BnAb; SOSIP (28); RRRRRR (53); and various other mutations described under “Results.”

A series of modified pcDNA3.1(−) vectors were constructed, each containing the CD5 secretion signal, a linker containing three alanines, and various Strep-tag II and octahistidine tags described under “Results.” Restriction sites NheI and NotI were introduced between the CD5 signal and the alanine linker. These plasmid vector DNAs isolated from 5-α competent *E. coli* cells (New England BioLabs, Inc.) were digested with NheI and NotI and dephosphorylated with FastAP alkaline phosphatase (Life Technologies).

The gp140 (and gp120) clones were constructed by either overlap extension PCR (56) or gene assembly PCR (57) using appropriate sets of primers. Restriction sites for NheI and NotI were introduced into the end primers. The amplified DNAs were digested with NheI and NotI and purified by agarose gel electrophoresis. The DNAs were then ligated with the NheI-NotI-digested and dephosphorylated pcDNA3.1(−) plasmid DNA. Directional insertion of gp140 DNA resulted in the in-frame fusion of gp140 with CD5 signal peptide at the N terminus and the alanine linker followed by various tags at the C terminus (see “Results”).

The gp140 (and gp120) clones were transformed into 5-α competent *E. coli* cells (New England BioLabs, Inc.), and the plasmid DNAs were purified using the GeneJET plasmid miniprep kit (Life Technologies). The DNAs were then sequenced to confirm 100% accuracy of the cloned gp140 DNA. For transfection into mammalian cells, the plasmid DNAs were purified using the GeneJET Plasmid Midiprep kit (Life Technologies) as per the manufacturer's instructions.

##### Small Scale Transfection

Suspension cells HEK293F (Life Technologies) and HEK293S GnTI^−^ (ATCC CRL-3022) were maintained in FreeStyle 293 expression medium (Life Technologies). The cells were incubated in a Multitron Pro shaker (Infors HT) at 37 °C in 8% CO_2_. In the case of HEK293S GnTI^−^, the growth medium was supplemented with 1% heat-inactivated fetal bovine serum (FBS, Quality Biologicals). For transfection, cells were grown overnight to a density of 1 × 10^6^ cells/ml. Two h prior to transfection, 6-ml cultures were centrifuged at 100 × *g* for 5 min and resuspended in fresh medium to a density of 2 × 10^6^ cells/ml in the absence of FBS. Three ml of cells were then transferred to each well of a 16.8-ml 6-well Clear Not-treated plate (Corning Inc.). For cleavage resistant (CR) (and gp120) DNA, 6 μg of gp140 plasmid DNA was added to the cells followed by the addition of linear polyethyleneimine (PEI25k, Polyscience Inc.) at a PEI/DNA (w/w) ratio of 3:1. For cleavage-proficient (CP) DNA, the cells were co-transfected with 3 μg of furin plasmid DNA. The cells were then incubated at 37 °C in 8% CO_2_ while shaking at 130 rpm overnight. After 12 h, 2 ml of fresh medium, 1 ml of HyClone SFM4HEK293 medium (GE Healthcare), and protein expression-enhancing sodium butyrate (58) solution (Sigma-Aldrich) to a final concentration of 2 nm were added to the cells. On day 5, the supernatant was harvested and clarified using a 0.2-μm filter (Corning).

##### Large Scale Transfection

Transfection was carried out in a manner similar to the small scale transfection, but it was scaled up to 1.2-liter cultures in a 2.8-liter flask and incubated at 37 °C in 8% CO_2_ while shaking at 90 rpm.

##### Small Scale gp140 Purification

To inactivate biotin present in the supernatant, 20 μl of Bio-Lock biotin blocking solution (IBA Life Sciences) was added to 5 ml of the supernatant containing the secreted gp140 (or gp120). After a 30-min incubation at 4 °C, 100 μl of Strep-Tactin beads (Qiagen) were added and allowed to rotate overnight at 4 °C. The bead mixture was spun down at 200 rpm to pellet the beads. The beads were then applied to a spin column (Pierce), briefly centrifuged to remove residual supernatant, and then washed twice with 50 mm Tris-HCl, pH 8, and 300 mm NaCl. The bound gp140 or gp120 proteins were eluted with 200 μl of Strep-Tactin elution buffer (2.5 mm d-desthiobiotin (Sigma), 25 mm Tris-HCl, pH 8, and 150 mm NaCl).

##### Large Scale gp140 Purification

To prevent nonspecific protease degradation, protease inhibitor tablets (Roche Diagnostics) were added to the clarified supernatant according to the manufacturer's instructions. To inactivate free biotin present in the culture medium, BioLock-biotin blocking solution (IBA Life Sciences) was added, and the medium was incubated at 4 °C for 30 min. The gp140 was purified by Strep-Tactin affinity chromatography followed by size exclusion chromatography (SEC). The supernatants were loaded onto a 1-ml Strep-Tactin column (Qiagen) at 0.7 ml/min using the ÄKTA Prime-Plus liquid chromatography system (GE Healthcare). Nonspecifically bound proteins were washed off by passing at least 20 column volumes of wash buffer (50 mm Tris-HCl, pH 8, and 300 mm NaCl) until the absorbance reached the baseline level. The Strep-tagged gp140 proteins were then eluted with elution buffer (2.5 mm d-desthiobiotin (Sigma), 25 mm Tris-HCl, pH 8, and 150 mm NaCl) at a flow rate of 1 ml/min. The peak fractions were pooled and concentrated using 100,000 molecular weight cut-off Amicon Ultra-4 centrifugal filter units (Millipore). The samples were then applied to a Hi-Load 16/600 Superdex-200 (preparation grade) size exclusion column (GE Healthcare) equilibrated with the gel filtration buffer (25 mm Tris-HCl, pH 8, 150 mm NaCl). Chromatography was done using the ÄKTA FPLC system (GE Healthcare), and fractions were collected and stored in 10% glycerol at −80 °C.

The gp140 clones fused to hexahistidine or octahistidine tags were purified by HisTrap affinity chromatography followed by SEC. The culture supernatant was loaded onto a 1-ml HisTrap HP column (GE Healthcare) at a flow rate of 0.7 ml/min using the ÄKTA Prime-Plus liquid chromatography system (GE Healthcare). Nonspecifically bound proteins were removed using a buffer containing 50 mm Tris-HCl, pH 8, 300 mm NaCl, and 20 mm imidazole until the absorbance reached the baseline level. The proteins were then eluted using a 20–500 mm imidazole gradient. The peak fractions were then applied to a Hi-Load 16/600 Superdex-200 (preparation grade) size exclusion column (GE Healthcare) and purified as described above.

##### SDS-PAGE and Blue Native PAGE (BN-PAGE)

SDS-PAGE analyses were performed using 4–20% gradient Tris-glycine gels (Life Technologies) or homemade 10% gels in the presence (reducing) or absence (non-reducing) of DTT. The BLUEstain protein ladder 11–245 kDa (Gold Biotechnology) was used as a molecular mass marker. BN-PAGE was performed using the Novex NativePAGE BisTris gel system in 4–16% gradient gels according to the manufacturer's instructions (Life Technologies). In the case of JRFL-FD, a native 4–12% gradient Tris-glycine gel (Life Technologies) was used with Tris-glycine buffer (Bio-Rad). The NativeMark unstained protein standard (Life Technologies) was used as the molecular mass marker. All gels were stained with Coomassie Blue R-250 solution.

##### Protease Cleavage

SEC-purified gp140 trimers were incubated with 10-fold serial dilutions (1–0.01 μg/ml) of Proteinase K (Thermo Scientific) at 37 °C for 1 h. The same preparation incubated at 37 °C without protease was used as a negative control. The samples were electrophoresed on reducing SDS gels for CR gp140 and non-reducing SDS gels for CP gp140.

##### Deglycosylation

For Strep-Tactin-purified gp140, 1 μl (500 units) of PNGase F (New England BioLabs, Inc.) was used to deglycosylate 10 μg of protein in the absence of DTT following the manufacturer's instructions. For SEC-purified trimers, deglycosylation was performed under native conditions using 3 μl (1,500 units) of PNGase F per 10 μg of protein and by incubating for 5 h at room temperature.

##### Strep-Tactin ELISA

Strep-Tactin-coated microplates (IBA Life Sciences) were coated with 1 μg/ml SEC-purified gp140 trimers in a volume of 100 μl/well of buffer (25 mm Tris-HCl, pH 7.6, 2 mm EDTA, and 140 mm NaCl) and incubated for 2 h at room temperature. Following three washes with PBST (0.05% Tween 20 in PBS), 100 μl of serially diluted Abs (10–0.001 μg/ml) in PBS were added to the wells, and the plates were incubated for 1 h at 37 °C. After three washes with PBST, the plates were incubated with 100 μl of rabbit anti-human Ab HRP conjugate (Santa Cruz Biotechnology) diluted 1:3,000 in PBS for 30 min at 37 °C. The plates were then washed three times with PBST, and the peroxidase substrate was added to develop the color reaction (TMB microwell peroxidase substrate system, KPL). The reaction was terminated by adding 100 μl of BlueSTOP solution (KPL), and OD_650_ was recorded using a VersaMax ELISA Microplate Reader (Molecular Devices).

##### Western Blotting

Polyclonal mouse Abs against HIV-1 JRFL gp140 were used as the primary Ab, and rabbit HRP-conjugated anti-mouse IgG (H+L) was used as the secondary Ab (Novex, Life Technologies). For Strep-tag II detection, StrepMAB-Classic HRP-conjugated Ab (IBA Life Sciences; dilution 1:1,000 in PBS) was used. Band intensities were measured using the Bio-Rad Gel Doc XR+ system and Image Lab software.

##### Negative Stain EM

Samples were diluted to 20–30 μg/ml and added to a glow-discharged carbon-coated grid. Samples were left on the grid for 2 min, blotted with a filter paper, and stained with Nano-W (Nanoprobes, Yaphank, NY) for 30 s, with two cycles of rinsing followed by stain application. After the last round of staining, the grid was blotted and allowed to dry completely before being imaged. Grids were imaged on an FEI Tecnai T12 microscope operating at 120 kV. Images were captured at a nominal magnification of ×67,000 on a Gatan UltraScan CCD using a dose of 20 electrons/Å^2^. Particles were selected semiautomatically using e2boxer within EMAN2 with a box width of 200 Å. Reference-free two-dimensional class averages were generated using EMAN2 (59). Briefly, several particles were manually picked to initiate automated particle picking using e2boxer within EMAN2. After automated particle picking, reference-free two-dimensional class averages were generated using e2refine2d within EMAN2. Each sample went through 15 iterations of two-dimensional classification, and 32 classes were generated per sample.

## Results

### 

#### 

##### Conventional Strategies Have Not Been Very Effective at Producing HIV-1 Env Trimers

**FIGURE 1.**
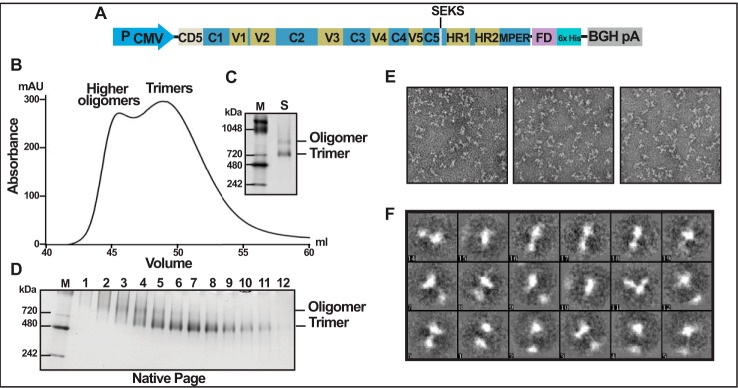
**Purification of uncleaved JRFL gp140 foldon trimers.** *A*, schematic of the JRFL foldon-gp140 recombinant construct showing the positions of the cleavage-resistant SEKS mutation, foldon, and His tag. *B*, elution profile of trimers and oligomers on SEC. *C*, native gel of starting material from HisTrap column that was loaded on SEC (*lane S*). *D*, native gel of the SEC fractions. *E*, negative stain EM of the peak trimer fraction from *B. F*, two-dimensional class averages of foldon trimers from *E*. The gels in *C* and *D* were stained with Coomassie Blue. *Lanes M*, *M*_r_ markers. The molecular masses in kDa of the marker proteins are shown on the *left*.

We have tested several codon-optimized gp140 constructs from HIV-1 strains JRFL, SF162 (clade B viruses), and CONPEP (clade C) for production of Env trimers. The gp140 DNA containing gp120 and gp41 ectodomain sequences truncated at aa 664 or 683 (HXB2 numbering) was cloned under the control of the CMV promoter (Fig. 1*A*) and transfected into a variety of mammalian cell lines (293F, 293T, 293EXPI, CHO, and GnTI^−^). With a signal peptide fused to the N terminus, gp140 was secreted into the culture medium, and the efficiency of production was quantified. Both CR and CP clones were tested. For CR gp140, the furin cleavage site REKR between gp120 and gp41 was mutated to SEKS, and for CP gp140, it was mutated to RRRRRR and co-transfected with a second furin-containing plasmid to enhance cleavage (53).

Of the three signal peptides tested, CD5, tissue plasminogen activator, and Gaussia Luciferase, CD5 showed consistently better expression. Fusing a hexahistidine (His) tag at the C terminus of gp140 did not affect expression, whereas an N-terminal tag showed poor expression probably because the hydrophilic tag affected cleavage of the adjacent, largely hydrophobic, signal peptide. However, the C-terminal tag bound poorly, if at all, to nickel-agarose.

Clones were also constructed by inserting the 27-aa phage T4 fibritin trimerization motif (foldon, or FD) (49) between the C terminus of gp140 and the His tag (*e.g.* JRFL; Fig. 1*A*). These produced a mixture of trimers and higher oligomers but *no* protomers (monomers or dimers). These, however, bound to nickel-agarose and could be further purified by SEC, which resolved the oligomers, but the elution profiles overlapped (Fig. 1, *B–D*). These results are consistent with the foldon-based trimers reported by other investigators (26, 60, 61) (also see Table 1). Further, our biochemical analyses showed that the protomers of these trimers are nonspecifically cross-linked with disulfide bonds (see below).

**TABLE 1:**
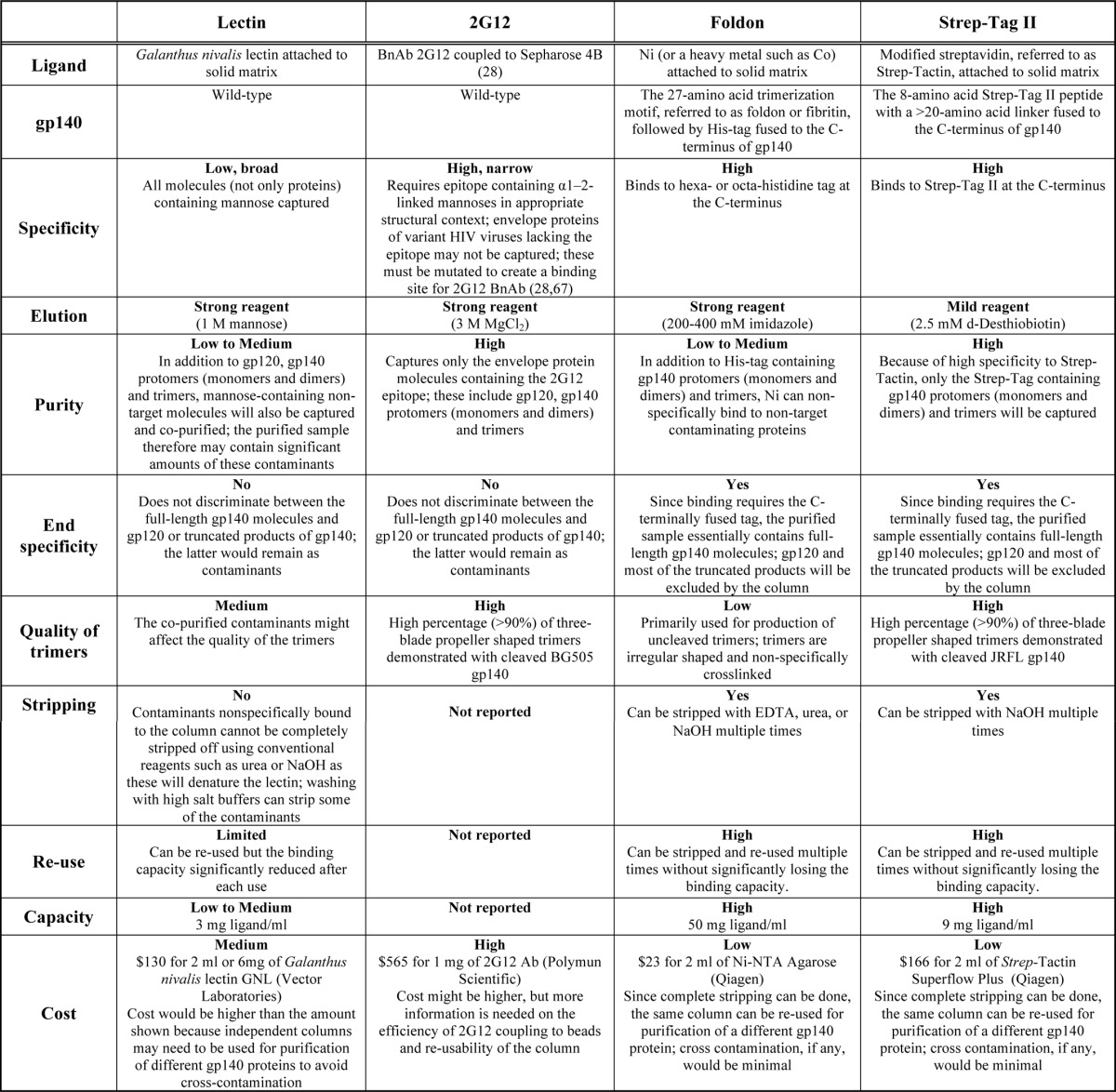
**Comparison of various approaches used for the purification of HIV-1 trimers**

We also constructed recombinants without any tag and captured gp140 using lectin beads (*Galanthus nivalis* lectin or concanavalin A). The protein was then purified by SEC. However, the yields of the trimers varied either by direct application of the culture supernatant or after 2–3× concentration by tangential flow filtration. We also encountered aggregation of some of the gp140 during lectin chromatography, and furthermore, the binding potency of lectin diminished progressively after each use.

Negative stain EM of trimers produced by the above approaches showed heterogeneous mixtures of particles. Relatively few were classic three-blade propeller-shaped, and some (e.g. the JRFL foldon trimers) showed variably shaped particles (Fig. 1, *E* and *F*), similar to that reported by Georgiev *et al.* (61), although all of these preparations behaved as “true” trimers by SEC and BN gel electrophoresis (Table 1).

##### Extended Strep-tag II Allows Efficient Isolation of gp140

We concluded that an approach that allows selective capture of gp140 directly from the culture medium would be most desirable for the production of trimers. Previous attempts to achieve this by fusing gp140 with a tag, such as the His tag, have failed. We hypothesized that these failures stemmed from the possibility that the tag, when attached to the base of the gp140 structure, was probably occluded, a problem further compounded by the presence of glycan shield, with up to 12 glycans attached to the C-terminal heptad repeat 2 (HR2) helices (see below). If our hypothesis was correct, extending the tag away from the base should make it more accessible for binding. The finding that the insertion of a 27-aa foldon sequence between the C terminus of gp140 and the His tag allowed efficient binding to nickel-agarose supported this reasoning.

**FIGURE 2.**
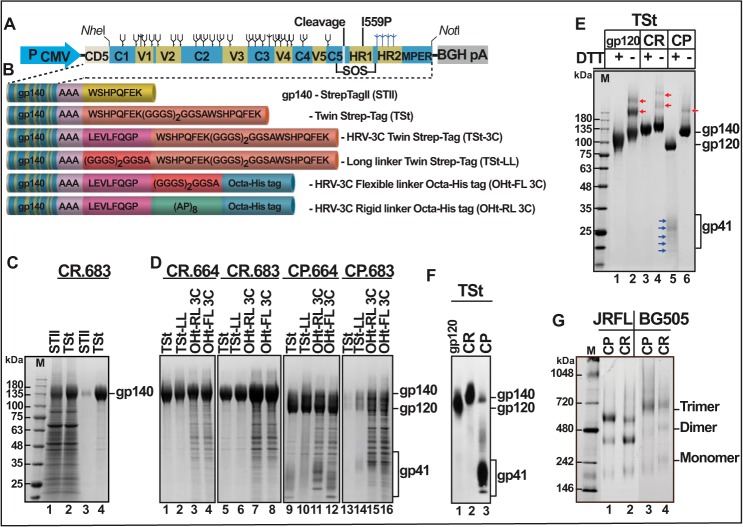
**Strep-tag II with an extended linker allows rapid purification of HIV-1 gp140 envelope trimers.** *A*, schematic of gp140 expression cassette. *P_CMV_*, CMV promoter; *CD5*, secretion peptide; *C1–C5*, conserved domains; *V1–V5*, variable domains; *black trees*, glycans in gp120; *blue trees*, glycans in gp41; *BGHpA*, 3′ poly-A sequence of bovine growth hormone gene. The positions of the furin cleavage site, disulfide bond mutations (*SOS*), and I559P mutation are shown. *B*, tags attached to gp140 C terminus. The aa sequence of each tag is shown in the *boxes*. HRV-3C refers to human rhinovirus protease cleavage site. *C*, and *D*, reducing SDS-polyacrylamide gels showing protein patterns of the samples as indicated at the *top. E*, SDS gel of samples under reducing (+*DTT*) or non-reducing (−*DTT*) conditions. *Red arrows*, oligomers of gp120 or gp140 formed by nonspecific disulfide cross-linking. *Blue arrows* correspond to the ladder of gp41 ectodomain bands glycosylated to varying extents. *F*, Western blot using Strep-tag II-specific mAb. *G*, BN gel of Strep-Tactin-purified gp140 samples. *Lanes labeled M*, *M*_r_ markers. The molecular masses in kDa of the marker proteins are shown on the *left*. Gels in *C*, *D*, *E*, and *G* were stained with Coomassie Blue.

We constructed a series of 36 recombinant clones by fusing the gp140 C terminus to Strep-tag II and octahistidine tag with various linkers in the middle (Fig. 2, *A* and *B*). Strep-tag II is an 8-aa peptide (WSHPQFEK) that binds to modified streptavidin, namely Strep-Tactin, at micromolar affinity and stringent specificity. However, the complex can be dissociated with desthiobiotin, a mild condition. We chose clade B JRFL gp140 as a template to evaluate this approach, but we also constructed, in parallel, clade A BG505 gp140 clones for comparison. Three “SOSIP” mutations and five “stabilizing” mutations were introduced to stabilize JRFL trimers (50, 51). The A501C and T605C mutations create an intraprotomer disulfide bond between gp120 and gp41, and the I559P mutation in the HR1 helix strengthens intersubunit (gp41) interactions, whereas the five mutations in or near HR1 (I535M, Q543L, S553N, K567Q, and R588G) strengthen gp120 and gp41 interactions at the interface (50–52).

Our data demonstrate that the Strep-tag II approach is highly effective to capture gp140 from the culture medium. Strep-tagged gp140 with a short Ala_3_ (or Gly-Ser-Gly-Ser) linker bound poorly to Strep-Tactin (Fig. 2*C*, *lane 3*), whereas the Twin Strep-tag containing 23-aa linker was efficiently captured (*lane 4*), although both clones expressed gp140 at similar levels (*lanes 1* and *2*). Bound gp140 could be specifically dissociated with 2.5 mm desthiobiotin, and the eluted protein was ∼95% pure (*e.g.* Fig. 2*D*, *lanes 1* and *2*). An HRV 3C protease cleavage site engineered between the gp140 C terminus and the linker was not cleaved, consistent with our hypothesis that a large protease molecule would encounter clashes with the protomer base. Various forms of gp140 could be efficiently captured (Fig. 2*D*): uncleaved (*lanes 1–8*) or cleaved (*lanes 9–16*); truncated at aa 664 (*lanes 1–4* and *9–12*) or aa 683 (*lanes 5–8* and *13–16*); tagged with octa-His with a flexible linker (*lanes 4*, *8*, *12*, and *16*) or a rigid linker (*lanes 3*, *7*, *11*, and *15*); clade A (BG505) and clade B (JRFL) viruses (Fig. 2*G*). In addition, we have also purified trimers from SF162 (clade B) and 40007 (clade CRF01 A-E) viruses^3^ (40007 gp140 sequence was kindly provided by Drs. Stovanabutra Sodsai, Jerome Kim, and Merlin Robb, HIV Medical Research Program, Walter Reed Army Institute of Research, Silver Spring, MD). Furthermore, unlike the lectin or 2G12 BnAb columns, our approach captures the full-length gp140 molecules and excludes gp120 and the truncated molecules that are often generated by nonspecific proteases (Table 1).

##### Strep-tagged JRFL gp140 Produces Abundant Amounts of Trimers

The CP and CR gp140 produced cleaved and uncleaved trimers, respectively. Cleavage by furin was nearly complete in CP gp140 (Fig. 2*E*, *lane 5*), whereas little or no cleavage was evident in CR gp140 (*lane 3*). The cleaved gp120 and gp41 subunits are covalently associated through the SOS disulfide bond, as evident from the appearance of a single 140-kDa band under non-reducing conditions (Fig. 2*E*, *lane 6*) and two bands (gp120 and gp41) under reducing conditions (lane 5). However, a ladder of five gp41 bands was also seen (*blue arrows* in lane 5), probably corresponding to glycosylation of 0–4 *N*-linked glycosylation sites (see below). The CR gp140, on the other hand, showed a single 140 kDa band under both reducing and non-reducing conditions (*lanes 3* and *4*) (the lack of cleavage of CR gp140 was further confirmed by Western blotting using a highly sensitive Strep-tag-specific mAb) (Fig. 2*F*). Whether the CR gp140 also formed the SOS bond could not be determined. Varying levels of higher oligomers were also seen in all preparations (including gp120), probably due to nonspecific disulfide cross-linking of the protomers under non-reducing conditions but much less so with the cleaved gp140 (Fig. 2*E*, *red arrows*, *lanes 2*, *4*, and *6*). About two-thirds of the Strep-tagged JRFL CP664-gp140 assembled into trimers (Fig. 2*G*, *lane 1*), whereas CR664-gp140 produced more dimers than trimers (*lane 2*). Similar patterns were also seen with Strep-tagged BG505 gp140. However BG505 produced higher levels of uncleaved trimers (*lane 4*, compare with *lane 2*), and the trimer bands were more diffused than JRFL, indicating more extensive glycosylation, as also evidenced by slightly higher *M*_r_ of these bands (*lanes 3* and *4*, compare with *lanes 1* and *2*).

##### Truncation of Cleaved gp140 beyond aa 664 Results in Poor gp140 Production

**FIGURE 3.**
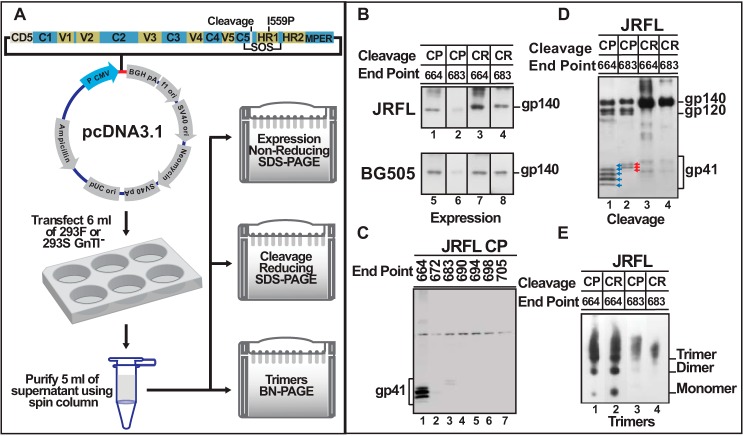
**Truncations beyond aa 664 produce few or no gp140 trimers.** *A*, a screening strategy to optimize recombinant gp140 production. *B*, *D*, and *E*, the secreted gp140 was captured by adding Strep-Tactin beads to the culture medium and eluted with 2.5 mm desthiobiotin. The same amount of the eluted sample was used to determine the efficiency of expression (*B*), cleavage and gp41 glycosylation (*D*), and trimer production (*E*). *C*, the efficiency of expression was further assessed by directly using the supernatant without the addition of Strep-Tactin beads. The samples were electrophoresed under reducing conditions so that gp41 would be cleanly separated and fully available for interaction with the Strep-tag mAb used for Western blotting. *B*, non-reducing SDS gel comparing the production of uncleaved and cleaved gp140 in the culture medium for aa truncations 664 and 683. *C*, reducing SDS gel comparing the production of gp140 recombinants truncated at various aa positions at the C terminus. End point numbers correspond to the aa at the end of the C terminus. The same volume of the supernatant was loaded in each lane. Note the presence of a nonspecific band in similar amounts in all of the lanes at about one-third of the distance from the *top* of the gel. *D*, reducing SDS gel of uncleaved and cleaved gp140 proteins truncated at aa 664 and 683. *Blue* and *red arrows*, differentially glycosylated gp41 ectodomain bands of gp140 proteins truncated at amino acids 664 and 683, respectively. *E*, BN gel of Strep-Tactin-purified gp140 samples. *B*, *D*, and *E*, Western blots using mouse anti-gp140 polyclonal antibody. *C*, Western blot using Strep-tag II-specific mAb. Data shown are for GnTI^−^-produced JRFL gp140. Similar patterns were observed for 293F-produced JRFL gp140 and for BG505 gp140 produced in GnTI^−^ or 293F cells.

We developed a rapid screening strategy to optimize various parameters for maximal trimer production, using any HIV-1 Env sequence (Fig. 3*A*). More than 40 different Strep-tagged gp140 clones were constructed, and each was transfected into a small volume (6 ml) of cells. The secreted gp140 was captured by adding Strep-Tactin beads directly to the culture medium, and the efficiency of expression (Fig. 3*B*), cleavage (Fig. 3*D*), and trimer production (Fig. 3*E*) was analyzed. The efficiency expression was further assessed by directly using the supernatant without Strep-Tactin purification (Fig. 3*C*). Different parameters tested include point of truncation, importance of SOSIP mutations and cleavage, production in 293F or GnTI^−^ cells (GnTI^−^ cells lack *N*-acetylglucosaminetransferase 1 and cannot introduce complex glycosylations), and clade B (JRFL) or A (BG505) gp140 (an *N*-glycosylation site was introduced into BG505 at aa 332 to make it equivalent to JRFL gp140) (62).

We observed similar patterns with both JRFL and BG505 gp140s expressed in 293F or GnTI^−^ cells. SOSIP mutations proved essential because without them, most of the protein aggregated and could not be captured by Strep-Tactin. Unexpectedly, however, we found that cleaved gp140 truncated beyond aa 664 produced lower amounts of gp140 in the culture medium (Fig. 3, *B* and *C*). The aa 672 and aa 683 constructs produced 3–5 times lower amounts, whereas further truncation resulted in nearly complete loss of gp140 production (Fig. 3*C*). This was not due to poor cleavage because 683-gp140 was efficiently cleaved, producing, as expected, slightly larger gp41 ladder bands (Fig. 3*D*, *lane 2*, compare with *lane 1*). In contrast, production of uncleaved 683-gp140 was not significantly affected. Unlike the cleaved 683-gp140, which was expressed at lower levels (Figs. 2*D* (compare *lanes 9–12* with *lanes 13–16*) and 3*B* (*lanes 2* and *6*)), the expression of uncleaved 683-gp140 was nearly as high as 664 (Fig. 3*B*, *lanes 3* and *7 versus lanes 4* and *8*). Finally, the aa 683 protein showed a tendency to aggregate, as shown by its appearance largely as a high *M*_r_ smear in the BN gel (Fig. 3*E*, *lane 3*).

The above results suggest that cleavage triggers a conformational change in the MPER, which might lead to exposure of some of the hydrophobic residues, leading to aggregation. This hypothesis is consistent with the previous reports by Klasse *et al.* (63) and Ringe *et al.* (26), which showed that the cleaved aa 681 (from KNH1144) and aa 683 (from BG505) gp140 proteins formed micelles at the MPER, presumably through interaction of the exposed hydrophobic residues of MPER with the lipid components.

##### Cleavage Is Essential for Production of Authentic HIV-1 Trimers

**FIGURE 4.**
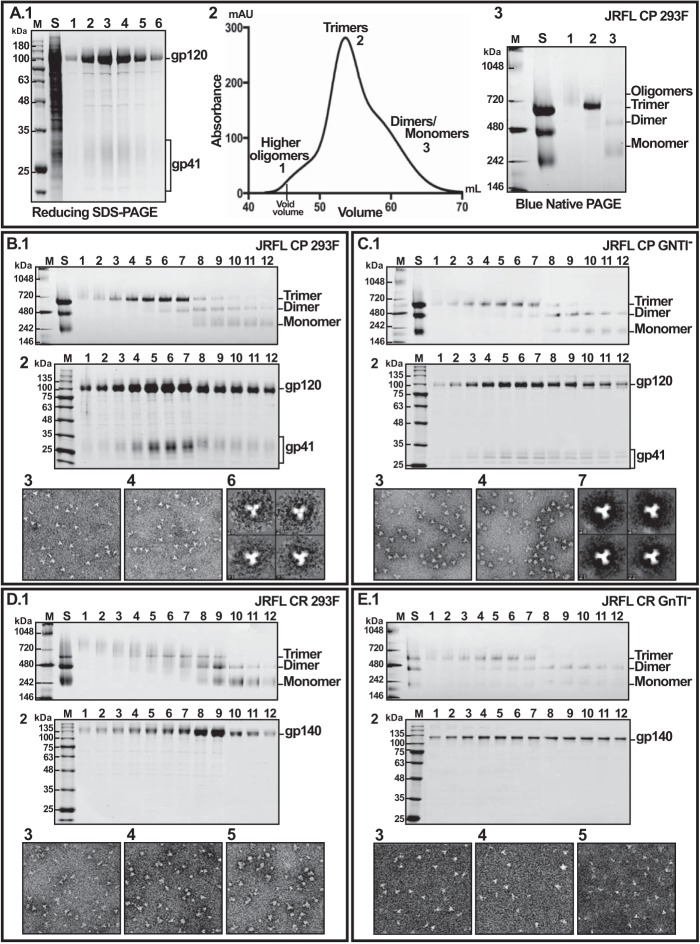
**Purification of cleaved and uncleaved JRFL gp140 trimers.** *A*, single-step purification of Strep-tagged gp140 from the culture supernatant by a Strep-Tactin column. *A.1*, reducing SDS gel of various gp140 samples. *Lane S*, culture supernatant; *lanes 1–6*, fractions eluted from Strep-Tactin column with 2.5 mm desthiobiotin. *A.2*, typical elution profile of gp140 oligomers from a Superdex 200 size exclusion column. *A.3*, BN gel of Strep-Tactin purified gp140 that was loaded on SEC (*lane S*) and three major fractions eluted from SEC (*lanes 1–3*, corresponding to *peaks 1–3* shown in *A.2. B*, purification of cleaved trimers expressed in 293F cells. *C*, purification of cleaved trimers expressed in GnTI^−^ cells. *D*, purification of uncleaved trimers expressed in 293F cells. *E*, purification of uncleaved trimers expressed in GnTI^−^ cells. The following applies to *panels* in *B–E. Panel 1*, BN gel of the SEC fractions. *Panel 2*, reducing SDS gel of fractions corresponding to those shown in *panel 1. Panels 3*, *4*, and *5*, negative-stain EM of the peak SEC fractions. *B.6* and *C.7*, reference-free two-dimensional class averages of trimers. The gels were stained with Coomassie Blue. *Lanes S*, starting material (Strep-Tactin-purified gp140) loaded on SEC. *Lanes M*, *M*_r_ markers. The molecular masses in kDa of marker proteins are shown on the *left*.

**FIGURE 5.**
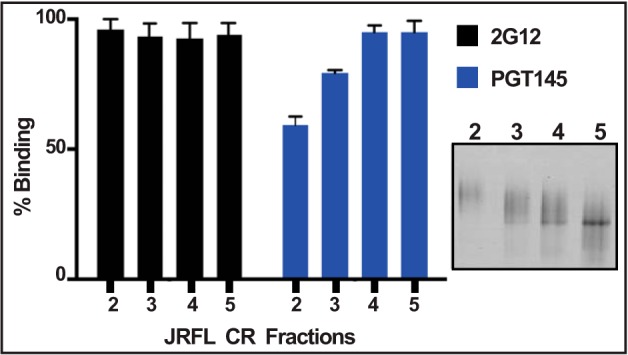
**Conformational heterogeneity of uncleaved trimers produced in 293F cells.** SEC fractions 2–5 from Fig. 4*D.1* were coated on Strep-Tactin plates at a fixed protein concentration of 1 μg/ml, and ELISAs were performed using the BnAbs 2G12 (*black bars*) or PGT145 (*blue bars*). *Inset*, Coomassie Blue-stained BN gel of fractions 2–5 depicting the presence of various amounts of smear in the fractions. The smear represents differential migration of conformationally heterogeneous trimers on the BN gel. Note the poor reactivity of fraction 2 containing an extensive smear to the conformation-specific PGT145 BnAb when compared with fraction 5 with a lesser smear. On the other hand, the 2G12 BnAb, which is not dependent on the conformation of the trimer, reacted equivalently to both fractions 2 and 5.

**FIGURE 6.**
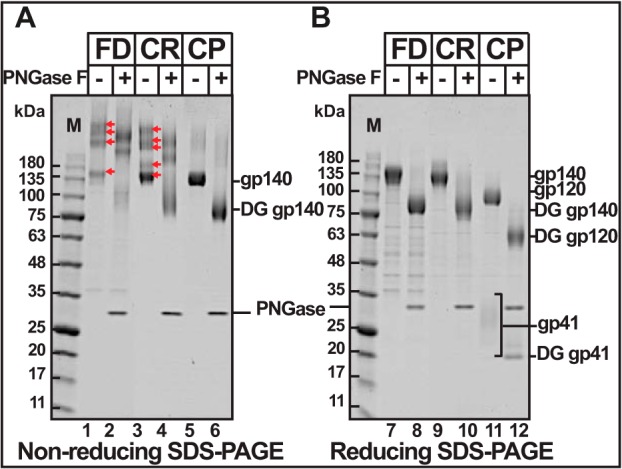
**The protomers of uncleaved trimers are nonspecifically cross-linked with disulfide bonds.** The SEC-purified JRFL trimers (Strep-tag uncleaved (*CR*), foldon-uncleaved (*FD*), and Strep-tag cleaved trimers (*CP*)) were electrophoresed under non-reducing (*A*) or reducing (*B*) conditions without (*lanes 1*, *3*, *5*, *7*, *9*, and *11*) or with (*lanes 2*, *4*, *6*, *8*, *10*, and *12*) PNGase F treatment. Note the presence of a ladder of high *M*_r_ bands in the FD uncleaved and Strep-tag uncleaved trimers (*red arrows* in *lanes 1* and *3*) but not in the cleaved trimers (*lane 5*). That these bands correspond to nonspecific disulfide cross-linked protomers, but not to differences in glycosylation, was shown by electrophoresis under reducing conditions and treatment with PNGase F. All of the high *M*_r_ bands were converted to a single band under reducing conditions (*lanes 7* and *9*), but the ladder remained after PNGase treatment (*lanes 2* and *4*), although the deglycosylated (*DG*) bands migrated faster due to the removal of glycans (when compared with *lanes 1* and *3*). The cleaved trimers did not show the high *M*_r_ ladder bands under non-reducing conditions (*lane 5*) and, as expected, converted to a faster-migrating DG band after PNGase F treatment (*lane 6*). Under reducing conditions, as expected, the cleaved trimers gave rise to gp120 and a ladder of gp41 bands (*lane 11*) and faster-migrating DG gp120 and single DG gp41 band (*lane 12*) after PNGase F treatment.

**FIGURE 7.**
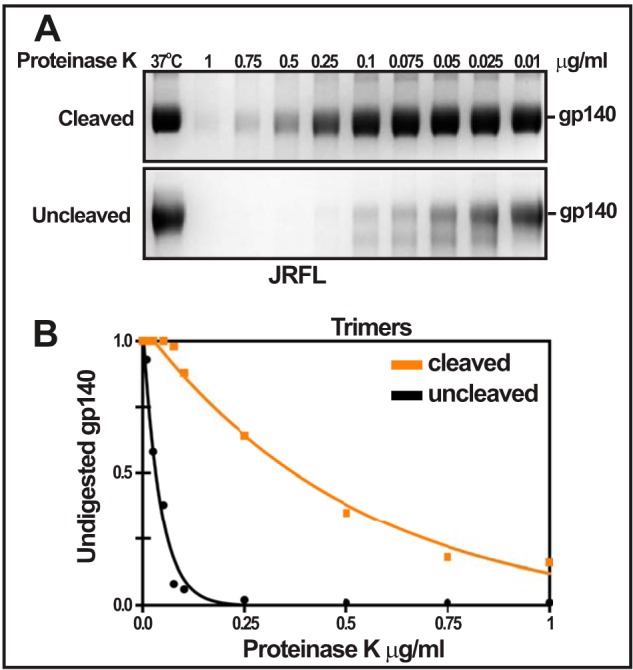
**Proteinase K sensitivity of cleaved and uncleaved JRFL trimers.** *A*, SEC-purified trimers from 293F or GnTI^−^ cells were treated with Proteinase K at the indicated concentrations for 1 h at 37 °C and electrophoresed on a reducing SDS gel followed by Coomassie Blue staining. The *37* °*C lane* corresponds to control sample incubated at 37 °C for 1 h without Proteinase K. Note that the uncleaved trimers are more susceptible to proteolysis than the cleaved trimers and also that the 293F trimers are more susceptible to proteolysis than the GnTI^−^ trimers. *B*, densitometric quantification of the undigested gp140 bands from *A*.

Strep-Tactin-purified gp140 was ∼95% pure (Fig. 4*A.1*), but it contained a mixture of trimers and protomers as well as some high *M*_r_ species (Fig. 4*A.3*, *lane S*). SEC separated these into three major fractions (Fig. 4*A.2*): (i) a high *M*_r_ fraction that eluted immediately after the void volume and migrated as a diffuse band on BN gel (*A.3*, *lane 1*); (ii) trimers, which eluted as a relatively sharp peak and migrated as a compact band on BN gel (*lane 2*); and (iii) two overlapping peaks of protomer dimers and monomers (*lane 3*). To determine which of the trimers were authentic, cleaved or uncleaved, 293F-produced (complex glycans) or GnTI^−^-produced (high mannose, no complex glycans) trimers were expressed on a large scale (1–4 liters) and purified by Strep-Tactin capture and SEC. The yields of gp140 were as follows: 293F CR, ∼20 mg/liter; CP, ∼12 mg/liter; GnTI^−^ CR, ∼3 mg/liter; CP, ∼1 mg/liter. Each SEC fraction was then analyzed by SDS-PAGE under reducing conditions to assess purity and cleavage (Fig. 4, *B*, *C*, *D*, and *E*) (*panels 2*), BN-PAGE to assess oligomeric state (*panels 1*), negative EM to assess the shape of the trimer (*panels 3–5*), and antigenicity to assess conformation (see below). These analyses showed that the trimers were purified to near homogeneity, up to 2–3 mg/liter (CP trimers), as well as establishing the criteria for their authenticity. First, we observed that in the case of JRFL Env, the fraction of gp140 recovered as trimers was 3–5-fold greater with cleaved gp140 than with uncleaved gp140 (compare *lanes S* and *5–7* of *B.1* with *D.1*). Second, the uncleaved trimers were of poor quality when compared with the cleaved trimers. Unlike the CP trimer fractions that showed a sharp band on the BN gel (*B.1*, *lanes 3–7*), the CR trimer fractions contained significant levels of diffused and high *M*_r_ species (*D.1*, *lanes 3–7*). The latter represented conformationally heterogeneous molecules, as was also evident from their poor reactivity with the conformation-specific BnAb PGT145 (Fig. 5). The reactivity was lowest with fractions containing the highest amount of these species. Third, the protomers of the uncleaved trimers as well as the foldon trimers were nonspecifically cross-linked through disulfide bonds, whereas the cleaved trimers showed much less cross-linking (Fig. 6). Fourth, the uncleaved trimers were more susceptible to nonspecific proteolysis, as evidenced by greater proteolysis of the CR trimers by Proteinase K than the CP trimers (Fig. 7). Finally, negative stain EM showed that the cleaved trimers appeared as three-blade propeller-shaped particles (Fig. 4, *B* and *C*, *panels 3* and *4*; reference-free two-dimensional class averages are shown in *panels B.6* and *C.7*), whereas the CR fractions showed fewer such particles (*D* and *E*; *panels 3–5*), and most were irregularly shaped. Overall, the above results are consistent with the behavior of the uncleaved and cleaved trimers generated by the 2G12 approach (see Table 1) (26).

##### Uncleaved Trimers Are Hyperglycosylated

**FIGURE 8.**
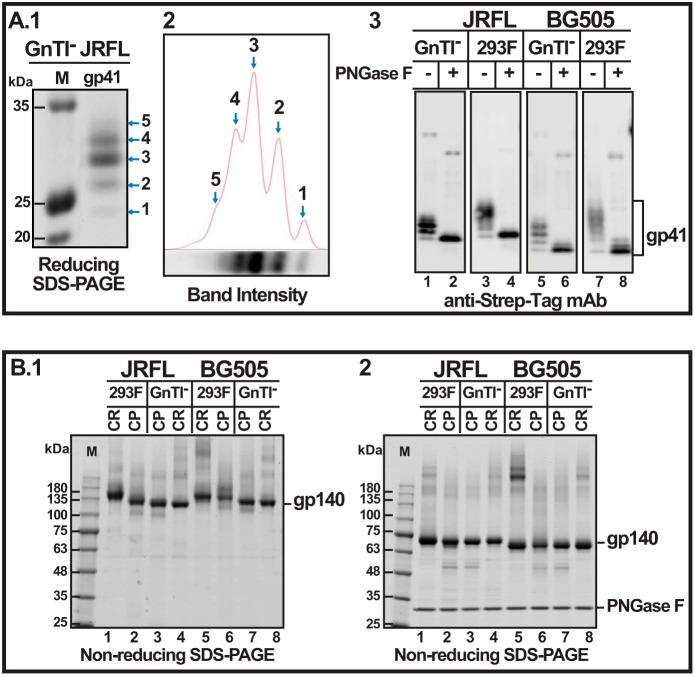
**Uncleaved trimers produced in 293F cells are hyperglycosylated.** *A1*, Western blot of reducing SDS gel using the Strep-tag II mAb showing the ladder of gp41 ectodomain bands. *A.2*, densitometric quantification of the intensity of the gp41 ladder bands shown in *A.1. A.3*, Western blot of reducing SDS gel using Strep-tag II-specific mAb. *B.1*, non-reducing SDS gel of uncleaved (*CR*) and cleaved (*CP*) JRFL and BG505 gp140 trimers produced in 293F or GnTI^−^ cells. *B.2*, non-educing SDS gel of samples from *B.1* after treatment with PNGase F. The gels were stained with Coomassie Blue. *Lanes M*, *M*_r_ markers. The molecular masses in kDa of marker proteins are shown on the *left*.

We observed that GnTI^−^ cells produced better quality trimers than the 293F cells, although the yields were lower in GnTI^−^ cells (Fig. 4, compare *panels* in *C* and *E* with the same *panels* in *B* and *D*). For instance, the diffused high *M*_r_ species described above were not seen in the CR trimers produced by GnTI^−^ cells (compare *D.1* and *E.1*, *lanes 1–7*). Negative stain EM showed a higher number of propeller-shaped trimers in the GnTI^−^-produced CR trimers than in the 293F-produced trimers (compare *panels 3–5* in *D* and *E*), which, in part, was due to heterogeneity in glycosylation. GnTI^−^ cells predominantly add Man5GlcNAc2, which is further processed by complex glycosylation in 293F cells. The presence of Strep-tag II at the C terminus of gp41 allowed evaluation of glycosylation using Strep-tag-specific mAbs. A ladder of five gp41 bands appeared when CPgp140 trimers were electrophoresed under reducing conditions. Of these, band 3 showed maximum intensity (Fig. 8, *A.1* and *A.2*). Because the JRFL gp41 ectodomain contains a cluster of four predicted *N*-linked glycosylation sites near its C terminus, these bands probably corresponded to glycosylation of 0–4 sites. This was confirmed by deglycosylation with PNGase F, which converted the ladder to a single species that migrated to the same position as the lowest band in the ladder, the unglycosylated gp41 (Fig. 8*A.3*, *lanes 2*, *4*, *6*, and *8*). Although a similar pattern was observed in both 293F and GnTI^−^ cells, the fully glycosylated 293F-gp41 bands were more diffused than the same from GnTI^−^-gp41 (Fig. 8*A.3*, compare *lanes 1–3* and *5–7*), presumably due to complex glycosylation. BG505 CP-gp140 showed similar banding patterns except that it appeared to undergo more extensive glycosylation. These results demonstrate “microheterogeneity” in gp41 glycosylation, albeit to a higher extent in 293F cells than in GnTI^−^ cells. Heterogeneity of gp41 glycosylation was also inferred in previous reports (64, 65).

The heterogeneity was more severe with the uncleaved trimers. Indeed, the uncleaved trimers produced by 293F cells were “hyperglycosylated.” Under non-reducing conditions, both the CP gp140 and CR gp140 were expected to migrate at the same position. They indeed did so when gp140 was produced by the GnTI^−^ cells (Fig. 8*B.1*, *lanes 3* and *4*). The 293F gp140 migrated slower than the GnTI^−^ gp140 (compare *lane 1* with *lane 4* and *lane 2* with *lane 3*), which was expected because gp140 undergoes complex glycosylations in 293F cells. Unexpectedly, however, the 293F-produced CR gp140 migrated slower than CP gp140 (compare *lanes 1* and *2*). Upon deglycosylation with PNGase F, all gp140 bands, whether uncleaved or cleaved, produced in 293F or GnTI^−^ cells, migrated at the same position (Fig. 8*B.2*). The same pattern was also observed with the BG505 gp140 tested in parallel (see *BG505 lanes 5–8* in Fig. 8). These results demonstrated that the 293F uncleaved trimers were hyperglycosylated when compared with their cleaved counterparts.

##### Antigenic Signatures Discriminate between Uncleaved and Cleaved Trimers

**FIGURE 9.**
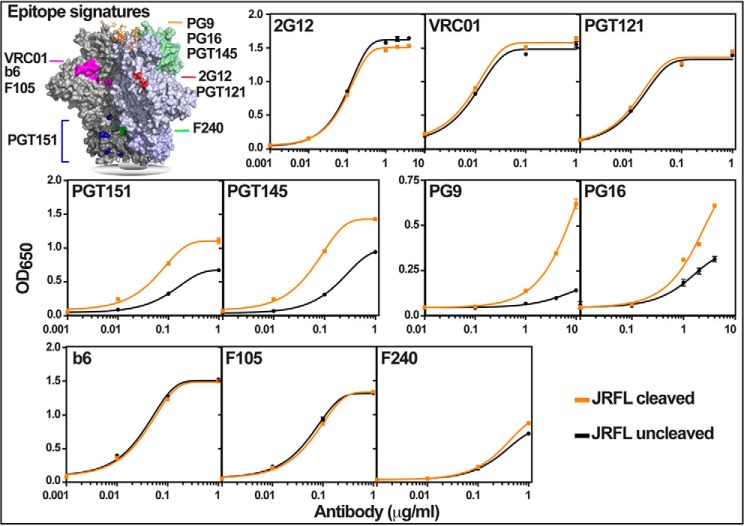
**Antigenic signatures of uncleaved and cleaved JRFL trimers.** The epitope signatures recognized by various Abs are shown in the three-dimensional context of the gp140 trimer structure (Protein Data Bank code 4TVP; the model was generated by PyMOL) (70, 81). These are *color-coded* and include amino acid residues as well as glycans. Purified cleaved and uncleaved gp140 trimers were coated on Strep-Tactin plates through the C-terminal Strep-tag II and incubated with various mAbs shown in the *top left corner* of each *panel*, and ELISAs were performed. The protein concentration of the trimers per well was kept constant at 1 μg/ml. Each *panel* shows the binding curve from three replicates at the indicated concentrations of the mAb. *Orange curves*, cleaved trimers. *Black curves*, uncleaved trimers. The *p* value as determined by the unpaired two-tailed *t* test is <0.05 for PGT151, PGT145, PG9, and PG16 at 1 μg/ml Ab. Repetition of ELISAs several times with independently purified trimers yielded similar results.

We have used an ELISA platform that can differentiate the structural and conformational states of cleaved and uncleaved trimers. Purified gp140 proteins were coated on Strep-Tactin plates through the C-terminal Strep-tag and incubated with mAbs that recognize different conformational signatures (Fig. 9). Because coating was done at neutral pH (unlike at pH 9 in traditional ELISAs), it would cause minimal, if any, structural perturbation. Moreover, the trimers were immobilized at a defined point; therefore, all immobilized molecules should be exposed in a similar orientation, in some ways mimicking the Env spikes displayed on the HIV-1 virion. Finally, the 23-aa flexible linker should make the trimer more accessible to Ab binding.

We first tested the reactivity of the trimers to BnAbs 2G12, VRC01, and PGT121. 2G12 recognizes a discontinuous epitope consisting of three or four high mannose glycans in the gp120 domain (66, 67). VRC01 is a potent BnAb that binds to the CD4 binding site and neutralizes >90% of the primary HIV-1 isolates (17). PGT121 primarily recognizes the complex glycan attached to Asn-332 (62). Consistent with the published data indicating that the conformational epitopes recognized by these Abs are well exposed in trimers as well as in gp120, both our cleaved and uncleaved trimers reacted strongly and equivalently to these Abs (Fig. 9) (23, 26, 28, 31, 68).

The BnAb PGT151 recognizes a conformational epitope containing aa residues and glycans present at the interface of gp120 and gp41 that is better exposed in the cleaved trimers (69, 70). Our results showed that the cleaved gp140 trimers exhibited stronger reactivity to PGT151 than the uncleaved trimers, suggesting that our cleaved trimers achieved native-like conformation.

The BnAbs PG16 and PG9 are quaternary Abs that neutralize 70–80% of the primary HIV-1 viruses. The quaternary specificity stems from their long hammerhead-shaped complementarity-determining region, which asymmetrically interacts with the V1, V2, and V3 loops of two protomers of the same trimer. The contact regions include, primarily, the V1V2 loop glycans Asn-160 and Asn-156/173, and residue Lys-168 of one protomer and glycans Asn-160 and Asn-197 (V3 loop) of the adjacent protomer (29, 71, 72). PGT145 is also a quaternary BnAb, however less well characterized, and it, like PG9 and PG16, recognizes the Asn-160 and Asn-156/173 glycans (42, 71). Consistent with the expectation that a compact trimer would react better with the quaternary Ab, the cleaved trimers reacted more strongly with PG9, PG16, and PGT145 BnAbs than the uncleaved trimers (Fig. 9).

Finally, the reactivity of our trimers with the non-neutralizing Abs (NnAbs) b6, F105, and F240 was tested. F105 and b6 recognize an epitope that includes the CD4 binding site, whereas F240 binds to the immunodominant loop of gp41 (aa 592–604) (16, 43, 73–75). Our CR and CP trimers as well as our protomers reacted similarly with these NnAbs, although the reactivity with F240 was poor overall, probably because its epitope is partially occluded (Fig. 9, *green* epitope signature).

Collectively, these data demonstrate that our trimers display antigenic signatures that are consistent with their cleaved or uncleaved states. Differential reactivity with the quaternary epitopes provides the best benchmark to ascertain the antigenic signature of compact, native-like trimers.

## Discussion

The trimeric envelope spike of HIV-1 virion makes the first contact with the host cell. It triggers fusion of viral and host membranes and delivers the nucleocapsid core into the cell. Trimer-specific Abs could disable Env function and block transmission of HIV. Development of a recombinant trimer immunogen, therefore, is one of the highest priorities in the hunt for an effective HIV vaccine (26, 32, 76). However, a myriad of variations reported in the literature led to confusion and controversy, and none could be effectively applied to diverse strains of HIV. For instance, recently, a procedure that produced native-like trimers from A-clade BG505 by using 2G12 BnAb to capture gp140 was not as effective with the B-clade JRFL trimers (26, 76). Hence, another procedure was developed in which lectin capture and negative selection by F105 NnAb was used to purify trimers (76). These Ab-based approaches have inherent limitations because the epitope signatures might vary from one HIV clade to another. In fact, it was necessary to mutate the wild-type BG505 gp140 in order to create the 2G12 binding epitope and allow for its purification by the 2G12 BnAb (26, 31). Here, we report a new approach that allows production of HIV Env trimers from potentially any HIV-1 clade or strain. We further present systematic analyses to optimize trimer production and biochemical characterizations to define the signatures of trimers.

A key feature of our approach is to selectively capture gp140 Env directly from the culture supernatant under mild conditions that cause minimal, if any, perturbation to the structure or oligomeric state of the protein. Attempts to achieve this using an affinity tag have thus far failed because the tag was not accessible for interaction with its binding partner. In accordance with the recent x-ray structures, the C-terminal aa 664 would not be accessible because it is the last residue of the long HR2 helices that encircle the base of the gp140 trimer (29, 70). It is further shielded by as many as 12 glycans emanating from these helices (29, 70). Therefore, it was essential not only to incorporate an exquisitely specific Strep-tag II but also to separate the tag from the base by a >20-aa-long linker. These modifications avoided clashes with the trimer base and allowed purification of nearly homogeneous protein in a single step. A variety of gp140 variants, cleaved, uncleaved, GnTI^−^-glycosylated, and 293F glycosylated from clades A, B, and A-E viruses, could be purified by this approach.

The Strep-tagged gp140 proteins behaved similarly to the native gp140. For instance, the CP gp140 was nearly completely cleaved to gp120 and gp41, and the CR gp140 remained uncleaved. SOSIP mutations were essential; otherwise, most of the gp140 aggregated into a high *M*_r_ fraction. Curiously, gp41 glycosylation was heterogeneous, showing five gp41 bands corresponding to glycosylation of 0–4 sites of the four *N*-linked glycosylation sites clustered in or near the 34-aa-long HR2 helix. This microheterogeneity, which was observed in both JRFL and BG505 gp140, might reflect a competition between the rate of glycosylation and the rate of folding of this transiently exposed structural element.

Our results show that cleavage is not essential for trimerization *per se*, but it is essential for maturation into propeller-shaped particles. Uncleaved gp140 produced such native-like particles but in fewer and variable numbers. Maturation might involve two, probably sequential, events, conformational transition and complex glycosylation (77). A cleavage-triggered conformational transition can be deduced from our experiments. Truncated CP gp140 constructs beyond aa 664 (*e.g.* aa 683) produced 3–5 times lower amounts of gp140, whereas the same truncation in a CR background was not significantly affected, and much of the aa 683 protein aggregated. Thus, the conformation of MPER where these residues are located must be different in the cleaved and uncleaved states. These results are consistent with the previous reports by Klasse *et al.* (63) and Ringe *et al.* (26), which showed that the cleaved aa 681 and aa 683 proteins formed micelles at the MPER. Perhaps some of the residues in the hydrophobic residue-rich MPER are better exposed in the cleaved state and associate with the membrane. Structural studies suggest that the MPER forms an L-shaped bent helix, and the residues 675–683 contact the virion membrane (78). Second, cleaved trimers exhibited greater stability and are less susceptible to proteolysis than the uncleaved trimers, suggesting that cleavage renders the trimers more compact and less accessible to protease. Finally, negative stain EM showed compact, propeller-shaped trimers in the cleaved state and irregularly shaped “blobs” in the uncleaved state, as was also observed by Ringe *et al.* (26) with the 2G12 produced trimers (Table 1).

Careful analysis of glycosylation patterns showed that cleavage channels trimers into the correct glycosylation pathway. Without cleavage, trimers from both JRFL and BG505 enter an aberrant pathway, resulting in hyperglycosylation, which traps the trimers in a loosely associated state. Consequently, the uncleaved trimers, including the foldon trimers produced by 293F cells, are conformationally heterogeneous, nonspecifically cross-linked, more susceptible to proteolysis, and irregularly shaped. The presence of a diffuse smear in the native gel, poor reactivity with the conformation-specific PGT145 BnAbs, and heterogeneity in gp41 complex glycosylations provide further evidence of this phenotype. Finally, the uncleaved trimers from GnTI^−^ cells that are unable to carry out hyperglycosylations showed a higher percentage of native-like trimers, further underscoring the negative effects of hyperglycosylation.

The strong reactivity of our CR and CP trimers with the BnAbs 2G12, VRC01, and PGT121 confirmed that the Strep-tagged trimers have correctly folded gp120 and gp41 ectodomains, exposing the respective conformational epitopes. Preferential reactivity of the cleaved trimers with the PGT151 BnAb further confirmed the integrity of the conformational epitope that emerges at the interface of gp120 and gp41 following cleavage. Strong reactivity with cleaved trimers, but not with uncleaved trimers, of the quaternary BnAbs PG9 and PG16 demonstrated that our CP gp140 protomers assembled into correct quaternary structure. Contrary to some reports that the CR trimers, but not the CP trimers, react with the NnAbs, both of our CR and CP trimers reacted similarly with the NnAbs b6, F240, and F105 (31). Careful examination of the published reports, however, showed that the reactivity depended on the type of assay platform used, and the sequences of the CR and CP trimers compared were not identical (Table 2). On the other hand, our data were generated using identical CP and CR sequences (except for the cleavage site), and our Strep-Tactin-based ELISA platform is not expected to introduce significant structural perturbations into the trimeric antigens. Furthermore, the HIV Env trimer is a dynamic structure and probably oscillates between “closed” and “open” states, allowing the NnAb to interact with the trimer when it opens transiently (79, 80). Thus, strong reactivity to the quaternary-specific BnAbs, such as PG9 and PG16, is the most reliable benchmark to assess the authenticity of the native-like trimers.

**TABLE 2:** **Reactivity of various cleaved and uncleaved trimers with non-neutralizing antibodies using different assay platforms** The table shows the reactivity of various cleaved (CP) and uncleaved (CR) trimer preparations with the non-neutralizing Abs b6, F105, and F240, using different assay platforms reported in the literature. Scores are assigned based on a comparison of the reactivity of different gp140 constructs reported in the same figure from each publication. Different publications were grouped into one line if the scores matched. Reactivity scores are shown as follows: +++, high; ++, moderate; +, weak; +/−, above baseline; −, negative. Note that the reactivity was dependent on the assay platform (compare the reactivity of BG505.SOSIP.R6.664 in ELISA *versus* SPR *versus* BLI) and the presence of the SOSIP mutation (compare the reactivity of BG505.SOSIP.SEKS.664 (line 4) *versus* BG505.WT.SEKS.664 by SPR (line 5)). Also, the ELISA data of BG505.SOSIP.R6.664 was compared with BG505.WT.SEKS.664 but not with its counterpart BG505.SOSIP.SEKS.664. NR, not reported.

Construct	Composition	Cleavage	b6	F105	F240	References
**ELISA**						
1. BG505.SOSIP.R6.664	Trimer	CP	+++	+	++	26, 28
2. BG505.WT.SEKS.664	Trimer	CR	+++	+++	+++	26

**SPR**						
3. BG505.SOSIP.R6.664	Trimer	CP	−/+	NR	−	26, 28, 31
4. BG505.SOSIP.SEKS.664	Trimer	CR	+	NR	−/+	26, 31
5. BG505.WT.SEKS.664	Trimer	CR	+++	NR	+++	26, 31

**Bio-layer interferometry**						
6. BG505.SOSIP.R6.664	Trimer	CP	++	−	NR	70
7. JRFL.SOSIP.R6.663	Trimer	CP	+++	++	NR	76*^a^*
8. 16055.SOSIP.R6.663	Trimer	CP	+++	++	NR	76*^a^*

*^a^* Before negative selection.

In conclusion, we have developed a new system to produce, optimize, and characterize pure and native-like HIV-1 Env trimers. Both cleavage and proper glycosylation are critical to generate compact, three-blade propeller shaped particles, whereas without cleavage, the trimers are heterogeneous in conformation, nonspecifically cross-linked, and hyperglycosylated, properties consistent with their irregular shape. The GnTI^−^ cells produced better quality trimers than the 293F cells. However, the 293F trimers might better recapitulate the native structure because GnTI^−^ cells lack complex glycosylations. The caveat, however, is that we do not know the glycan structures introduced by the 293F cells and whether these are the same as that present on the HIV-1 virion. Microheterogeneity of glycosylation might also be a concern. Three criteria, namely ≥95% cleavage, nearly 100% propeller-shaped particles, and strong reactivity to quaternary BnAbs, define authentic HIV-1 trimers. We believe that the Strep-tag approach provides several useful features (Table 1) and is broadly applicable to generate trimers from potentially any HIV-1 virus for basic research as well as for human clinical trials and vaccine manufacture. The well behaved JRFL trimers described here might serve as a good scaffold for further engineering to generate a trimeric immunogen that can elicit transmission-blocking Abs against diverse HIV-1 strains.

## Author Contributions

W. A., C. H., D. F., and G. G. constructed the recombinants and did the cell culture work. W. A., M. M., and N. A. purified proteins. W. A. did glycosylation studies, and M. M. did the ELISA work. V. B. R. designed the research and wrote the manuscript with the assistance of W. A., N. A., and M. M.

## Footnotes

3 N. Ananthaswamy, W. Alsalmi, M. Mahalingam, and V. B. Rao, manuscript in preparation.

## Footnotes

2 The abbreviations used are:

Ab: antibody
BnAb: broadly neutralizing Ab
Nn: non-neutralizing Ab
HR1 and HR2: heptad repeats 1 and 2, respectively
MPER: membrane-proximal external region
aa: amino acid(s)
SEC: size exclusion chromatography
BN: blue native
BisTris: 2-[bis(2-hydroxyethyl)amino]-2-(hydroxymethyl)propane-1,3-diol
PNGase F: peptide:*N*-glycosidase F
CR: cleavage-resistant
CP: cleavage-proficient
FD: foldon a bacteriophage T4 fibritin trimerization motif.
